# Nutritional Assessment of the Symptomatic Patient on a Plant-Based Diet: Seven Key Questions

**DOI:** 10.3390/nu15061387

**Published:** 2023-03-13

**Authors:** Gregory A. Plotnikoff, Linda Dobberstein, Susan Raatz

**Affiliations:** 1 Minnesota Personalized Medicine, Minneapolis, MN 55403, USA; 2 Department of Food Science and Nutrition, University of Minnesota, Minneapolis, MN 55455, USA

**Keywords:** plant-based diet, vegan, vegetarian, vitamin D, vitamin B12, iron, essential fatty acids, calcium, zinc, essential amino acids

## Abstract

Plant-based diets, both vegan and vegetarian, which emphasize grains, vegetables, fruits, legumes, nuts, and seeds are increasingly popular for health as well as financial, ethical, and religious reasons. The medical literature clearly demonstrates that whole food plant-based diets can be both nutritionally sufficient and medically beneficial. However, any person on an intentionally restrictive, but poorly-designed diet may predispose themselves to clinically-relevant nutritional deficiencies. For persons on a poorly-designed plant-based diet, deficiencies are possible in both macronutrients (protein, essential fatty acids) and micronutrients (vitamin B12, iron, calcium, zinc, and vitamin D). Practitioner evaluation of symptomatic patients on a plant-based diet requires special consideration of seven key nutrient concerns for plant-based diets. This article translates these concerns into seven practical questions that all practitioners can introduce into their patient assessments and clinical reasoning. Ideally, persons on plant-based diets should be able to answer these seven questions. Each serves as a heuristic prompt for both clinician and patient attentiveness to a complete diet. As such, these seven questions support increased patient nutrition knowledge and practitioner capacity to counsel, refer, and appropriately focus clinical resources.

## 1. Introduction

In recent decades, both practitioners and patients have grown in awareness of the potentially life-saving importance of shifting to a plant-based diet [1,2]. Several systematic reviews and meta-analyses of vegan or vegetarian diets have demonstrated benefits for reduced blood pressure, improved glycemic control, reduced total and LDL cholesterol, reduced pain from diabetic neuropathy, reduced C-reactive protein, as well as reduced weight [3,4,5,6,7,8,9]. With such evidence, plant-based diets are widely promoted and increasingly prescribed by numerous health care practitioners [10,11].

The definition of a plant-based diet itself is the subject of some confusion in the medical literature. A recent review documented that the majority of plant-based dietary intervention studies completely proscribed animal products. However, more than 30% of trials included dairy products and 20% were semi-vegetarian [12].

In the 12-million-member Kaiser-Permanente health plan (United States), the patient-directed Plant-Based Eating Guidebook (see Table 1) describes a plant-based diet as “lots of plant foods in their whole, unprocessed form, such as vegetables, fruits, beans, lentils, nuts, seeds, whole grains, and small amounts of healthy fats. It does not include animal products, such as meat, poultry, fish, dairy, and eggs. It also does not include processed foods or sweets” [11]. The formal definition in the medical literature is that a plant-based diet consists of all minimally processed fruits, vegetables, whole grains, legumes, nuts and seeds, herbs, and spices; and excludes all animal products, including red meat, poultry, fish, eggs, and dairy products [13].

Ideally, people on a plant-based diet will follow a well-planned diet that provides all the necessary nutrients and energy for health. Realistically, however, a well-designed plant-based diet may not be well-implemented [14]. A plant-based diet is an exercise in mindfulness. As with any diet, in the absence of mindful attention to dietary choices, key nutrients may be missing [15,16,17,18,19,20,21].

Numerous reflexive factors guide dietary choices and require mindful attention. These include culture, upbringing, and comfort with cooking, access, convenience, cost, as well as available time or energy. Additionally, plant-based diets can include highly-processed foods that increase health risk including refined grains, fried foods, and added sugar that increase nutritional inadequacy and health risk [11].

A healthy plant-based diet may be further complicated by additional restrictions, such as gluten-free diets or diets that minimize ingestion of lectins, corn, histamine, tyramine, methionine, oxalates, salicylates, nickel, mold, or FODMAPs. Even more nutritional challenges are found in patients with hypochlorhydria [22]; food intolerances; renal insufficiency [23] malabsorption syndromes (including those who have undergone gastric bypass or cholecystectomy); [24] or prescribed metformin, proton pump inhibitors, or H2 blockers [25].

Furthermore, pregnancy, breastfeeding, childhood/adolescence, intense physical work, or athletic training represent additional challenges for a healthy diet. Based upon decades of clinical experience, innumerable common patient scenarios exist where the nutritional adequacy of a plant-based diet needs to be considered in the clinical assessment of symptomatic patients. To the extent that nutrition is important for health, the differential diagnosis for all patients with many common, non-specific symptoms, including fatigue, depression, anxiety, insomnia, dizziness, cognitive complaints, as well as chronic pain includes the possibility of one or more dietary nutrient deficiencies [26,27]. Ascertainment of what types of foods are preferred or omitted provides guidance as towards adequacy or inadequacy of nutrient status.

Just as with any other prescribed intervention, the monitoring and reassessment of a prescribed plant-based diet is medically indicated. However, patients may also embark on a plant-based diet with neither a prescription nor sufficient guidance. They may add other dietary restrictions or be unaware of increased risks with prescribed medications. Ironically, this means that well-intentioned, but poorly-designed or poorly-implemented diets for better health may actually promote nutritional deficiencies.

For all these reasons, practitioners now need the capacity to assess quickly if their patient is following a well-planned, plant-based diet. This is especially true for the patient with symptoms presenting for diagnosis. Yet, numerous studies have documented that physicians may lack both the knowledge and the confidence to address nutritional concerns [28,29,30,31,32,33,34].

Additionally, a recent review of the food-based dietary guidelines for 100 countries found that most countries do not provide information to their citizens that cover the broad spectrum of plant-based diets [35]. To address these concerns, the authors in this paper seek to increase clinician knowledge and to affirm the importance of referrals to dietitian nutritionist colleagues.

Seven nutrients require special consideration. For patients on any form of a plant-based diet presenting for assessment of new symptoms or worsened chronic symptoms, physicians need to consider their intake of vitamin B12, iron, essential fatty acids, calcium, vitamin D, zinc, and protein. To rapidly screen in primary or subspecialty care, we identified the following seven questions as practical guides that can empower physicians to both consider nutritional status in their clinical reasoning and appropriately utilize clinical resources.

## 2. Question One: What Is Your Preferred Source of Vitamin B12?

Dietary vitamin B12 for humans is only produced by the microbial and archaea kingdoms. This vitamin is readily found in dairy, eggs, meat, and fish as animals can concentrate this nutrient and feedlot animals are often supplemented.

Persons on a plant-based diet without vitamin B12 supplementation, as well as elderly or pregnant persons, are more susceptible to B12 deficiency [36]. Plant-based diets are high in folate (vitamin B9), which can mask B12 deficiencies until problems develop [37].

### 2.1. Patient Proficiency

Unfortified plant-based diets do not contain bioactive vitamin B12 (cobalamin). Vitamin B12 is the most common vitamin deficiency in the vegan diet [16].

People on plant-based diets require vitamin B12 supplementation. The only reliable plant-based sources are processed foods fortified with vitamin B12. Examples include fortified plant milks, breakfast cereals, soy products, and nutritional yeasts. Fermented soy products (e.g., miso, tempeh), shiitake mushrooms, algae, and unfortified nutritional yeast contain inactive analogues of vitamin B12 can contribute to vitamin B12 intake, but are not considered reliable sources [38,39]. The dried seaweed known as nori has been shown to provide active vitamin B12 [40]. Low vitamin B12 can adversely affect mood, memory, energy, and nerve function [41,42]. Persistently low B12 can result in irreversible neurocognitive dysfunction [41,42].

### 2.2. Practitioner Proficiency

Vitamin B12 is the necessary co-factor for only two enzymatic reactions in human physiology [43,44,45,46]. Despite this, an incredibly broad array of symptoms can follow from B12 insufficiency.

The first enzyme, methionine synthase, is crucial in the methylation cycle. Insufficient B12 in the methylcobalamin form results in elevated homocysteine and impairment of all methylation-dependent processes including [46,47]:

- Neurotransmitter metabolism (mood, energy, cognition);
- Estrogen metabolism (PMS, endometriosis, menstrual cycle irregularities);
- Histamine clearance (histamine intolerance);
- Phosphatidylcholine production (cell membrane composition and function, intestinal mucus, pulmonary surfactant);
- Creatine and carnitine production (cognition, strength/stamina, mood);
- Myelin production (neurologic, cognitive function);
- DNA synthesis.

The second enzyme, L-methylmalonyl-coA mutase, is the last enzyme needed for fatty acids and amino acids to enter the Kreb’s cycle (via succinyl CoA rather than acetyl CoA). Insufficient B12 in the adenosylcobalamin form results in elevated methylmalonic acid [46,48,49].

For women of childbearing age, B12 status is important for both maternal health as well as minimizing neural tube defect risk in the child [50]. In infants, maternal and infant vitamin B12 status is relevant for normal feeding, gastrointestinal function, growth, and neurological development [51]. In adults, common, non-specific symptoms of B12 deficiency include fatigue, nausea, anorexia, arthralgia, insomnia, dyspnea upon exertion, dizzy spells, feeling cold, pallor, muscle cramps, and digestive complaints. Additional deficiency signs are neurologic, cognitive, and psychiatric. Neurologic signs include paresthesias, sensory loss, ataxia, neuropathies including ocular neuropathy, age-related macular degeneration, autonomic dysfunction including urinary incontinence, orthostatic intolerance, excessive sweating and erectile dysfunction, plus motor disorders, cerebral atrophy, and spinal cord degeneration [43,49]. Cognitive signs include word finding and concentration difficulties, disorientation, and dementia. B12-dependent psychiatric disorders include depression, postpartum depression, and psychoses [52,53,54].

B12 absorption in the terminal ileum requires stomach hydrochloric acid and pepsin, as well as the sequential binding of three transport proteins: haptocorin (saliva and stomach), intrinsic factor (stomach and intestine), and transcobalamin (intestine into liver and systemic circulation). Risk factors for poor absorption include auto-antibodies to intrinsic factor and/or parietal cells, impaired acid production, *Helicobacter pylori* infection, gastric bypass, intestinal bacteria overgrowth, and malabsorption disorders. Iatrogenic risk factors for low vitamin B12 absorption include use of proton pump inhibitors or metformin [55]. Oral B12 may not be readily assimilated in patients with one of several common genomic variations [49,56]. B12 deficiency can occur in the absence of macrocytic anemia and when serum B12 level is within the reference range [43].

Cyanide-stabilized cobalamin, cyanocobalamin, is the most common B12 supplement [50,57,58,59]. Cyanocobalamin releases a cyanide group for every molecule of B12 that is used. Theoretically, this could be important for persons with diets rich in cyanide via the ingestion of almonds, lima beans, soy, spinach, and seeds; who smoke; are uremic; or have the most common of inherited mitochondrial disorders, Leber hereditary optic neuropathy [59,60]. Options include oral or sublingual methylcobalamin, adenosylcobalamin, or hydroxocobalamin. Daily oral doses at 1000 µg (far above the RDA of 2.4 µg/day) have shown equivalence or superiority to injected B12 at 1000 mcg/month [52].

There are no clearly defined adverse effects produced by vitamin B12 supplementation. A no tolerable upper intake level (UL) has been established for B12, due to its low level of toxicity [61]. Compounded methylcobalamin is available for intramuscular administration.

### 2.3. Clinical Testing Notes

Vitamin B12 deficiency can be easily missed. The absence of macrocytic anemia does not rule out significant B12 deficiency [43,53,62]. Additionally, measuring only a serum B12 level has poor sensitivity for identifying early insufficiency [43,63,64,65]. The medical literature does not support a clear vitamin B12 cut-off for diagnosis [43,66,67,68]. Evaluation of vitamin B12 status should include at least one systemic biomarker (serum B12 or holotranscobalamin) and one cellular biomarker (homocysteine or methylmalonic acid, MMA). Optimal testing for vitamin B12 needs to be conducted after 12 h of fasting and no B12 supplementation for at least one day. Of note, normal levels of serum B12, homocysteine, and MMA still may not exclude symptomatic B12 deficiency. Clinical suspicion must remain high [49,65,67,68,69]. Treatment trials may be helpful for diagnosis [43,69].

Of potential relevance to testing patients not supplementing, B12 may be an acute phase reactant. Measured B12 levels may be artificially elevated in myeloid, lymphatic, and solid tumors, alcoholism, cancer, liver-, renal-, autoimmune-, and bronchopulmonary diseases [41].

## 3. Question Two: What Is Your Preferred Source of Iron?

Iron deficiency is the most common cause of anemia across the globe, affecting one-third of the world population [70]. About 10 million Americans are iron deficient and 5 million have iron deficiency anemia [71]. Iron found in meats (heme iron) has a higher bioavailability than iron found in plants (non-heme iron) [72]. Moderately lower iron stores may reduce the risk of chronic diseases [73], but may also increase the risk of restless leg syndrome [71].

### 3.1. Patient Proficiency

Depleted iron stores and iron-deficiency anemia are commonly found in all persons regardless of diet [70,71]. However, persons on plant-based diets, especially women of child-bearing age, children, and teenagers need to be aware of an increased risk. Iron status is important for optimal mood, energy, cognition, athletic performance, and pregnancy outcomes [2,71,74,75,76,77,78].

Plant-based sources of iron include Swiss chard, spinach, quinoa, soybeans, sesame, pumpkin seeds, lima beans, lentils, tempeh, tofu, cashews, almonds, blackstrap molasses, and iron-enriched baked goods such as bagels. Vitamin C aids iron absorption. Good sources of vitamin C include citrus fruit, red and green peppers, broccoli, and tomato products; in addition, Kiwi spices such as anise, caraway, cumin, licorice, and mint also promote iron absorption [79].

Other food, spices, beverages, and supplements may impair iron absorption [80]. These include rosemary, chili pepper, garlic, Pak hyeng, shallot, tamarind, soy beans, milk, eggs, coffee, green and black tea, as well as turmeric, calcium, resveratrol, and quercetin [73,79,81].

### 3.2. Practitioner Proficiency

Iron is an important co-factor for the synthesis of hemoglobin, myoglobin, neuroglobin, nitric oxide, dopamine, and DNA. Iron is also an important co-factor as well as for oxidative stress management (superoxide dismutase), DNA repair, and mitochondrial function (aconitase, cytochrome c, cytochrome c oxidase) [82]. As a consequence, iron is important for cognitive performance, cardiac function, gastric digestion, muscle strength, endurance, and stamina, in addition to temperature regulation. Normal iron status is important during pregnancy for fetal brain maturation and optimal birth weight, as well as for the prevention of adverse maternal outcomes including mortality. Low-iron status (Ferritin levels less than 50 µg/L) is the leading cause of restless legs syndrome [71,71]. Overt and prolonged iron deficiency eventually results in reduced hematocrit, hemoglobin, and RBC levels.

Dietary phytates and tannins [83], malabsorption [84], infections, small intestinal bacteria overgrowth (SIBO) [85], digestive inflammation, as well as systemic inflammation [86] can impair iron absorption even with adequate dietary intake. Blood loss, blood and platelet donations, and rapid growth contribute to iron deficiency [71,71]. Gastric acid is necessary for non-heme iron to dissociate and increase its solubility for absorption. Acid-blocking medications and hypochlorhydria disrupt iron absorption [87]. A normal hemoglobin does not exclude iron deficiency. Iron stores significantly influence non-heme iron absorption [88,89].

### 3.3. Clinical Testing Notes

Serum ferritin is the primary indicator for iron deficiency. This test is the most efficient and cost-effective means for diagnosis. Ferritin levels less than 15 µg/L are diagnostic. Erythropoiesis may be affected even at higher levels of 40 µg/L [90].

## 4. Question Three: What Is Your Preferred Source of Essential Fatty Acids?

The short-chain omega-3 and omega-6 fatty acids, alpha-linolenic acid, and linoleic acid are termed essential as they cannot be synthesized and must be ingested for multiple biological functions [91,92,93].

The activity of their elongated long-chain forms (omega-3: eicosapentaenoic acid (EPA, C20:5n3) and docosahexaenoic acid (DHA, C22:6n3); omega-6: DGLA (dihomo-gamma-linoleic acid, cis-20:3n6); and AA (arachidonic acid 20:4n3) include optimal cell membrane composition, inflammatory and anti-inflammatory response (production of eicosanoids: prostaglandins, leukotrienes, and thromboxanes), inflammation resolution (production of specialized pro-resolving mediators (SPM) including lipoxins, resolvins, maresins, and protectins), as well as endocannabinoid production [94,95,96].

The essential fatty acids were originally termed “vitamin F” when identified as essential factors for growth and dermal function in 1929 [97,98]. The long-chain fatty acids were identified when it was discovered that the essential fatty acids linoleic and linolenic acid gave rise to the pentaene and hexaene acids, EPA and DHA of rat tissue [99].

### 4.1. Patient Proficiency

Essential nutrients means that we must eat them; we cannot make them. The short-chain omega-3 and omega-6 fatty acids, alpha-linolenic acid and linoleic acid, are termed essential as they cannot be synthesized and must be ingested for multiple biological functions [100,101]. Two families of essential fatty acids exist: the omega-3 and omega-6 fatty acids. Optimal plant-based short-chain omega-3 oils are found in walnuts, flaxseeds, hemp seeds, and chia seeds. Optimal dietary omega-6 sources include cold-pressed safflower, sunflower, and olive oils; plus avocados, nuts, and seeds [93,102].

Long-chain omega-3 fatty acids (termed EPA and DHA) are vitally important and can be produced from short-chain omega-3′s with the help of several vitamins and minerals; but the process may be inadequate. Persons on plant-based diets are at increased risk for insufficient EPA and DHA [103].

Low EPA or DHA levels can adversely affect mood, memory, and inflammation/pain in addition to infant development [104]. Algae-derived DHA or brown algae with kelp oil may support long-chain omega-3 intake in plant-based diets [105].

### 4.2. Practitioner Proficiency

Essential fatty acids needs can be met by a plant-based diet, but sufficiency requires planning and adequate co-factors [103,106]. The short-chain omega-3 fatty acid termed alpha linoleic acid (ALA) can be converted into long-chain fatty acids omega-3 EPA and DHA; but the process may be insufficient with a low conversion rate, especially for men [102,107,108,109]. Insufficient iron, zinc, magnesium, vitamins C, and B6 intake also contribute to poor conversion rates, which may substantially impact infants, children, and women [106,110,111].

Low EPA levels are associated with inflammatory imbalances, autoimmune diseases, arthritis, asthma, atherosclerosis, menstrual cramps, eczema, psoriasis, depression, and attention deficit disorders [106,110,112,113].

Maternal DHA intake and supply is crucial in the third trimester and during breastfeeding for an infant’s neurological development and function. DHA regulates levels of neurotrophins, i.e., brain-derived neurotrophic factor (BDNF) and nerve growth factor. At all ages, cell membrane DHA content determines membrane fluidity and hormone- receptor binding capacity. Low DHA levels are associated with ADHD, cognitive dysfunction, depression, diabetes, hyperestrogenism, aggression, and impulsive violence including suicide. Additional concerns of low DHA include gestational diabetes, hypertension, pre-eclampsia, premature birth, low birth rate, post-partum depression, post-partum obsessive-compulsive disorder, menopausal problems, osteoporosis, breast cancer, and cardiovascular disease [103,105,110].

## 5. Question Four: What Is Your Preferred Source of Calcium?

Sufficient intake and absorption of calcium is essential for ensuring normal bone and dental health. Dairy is the best-known dietary calcium source, but is intentionally not included in plant-based diets. Persons on plant-based diets may be at even higher risk than the general public for insufficient dietary calcium intake [2]. To ensure normal blood levels, dietary calcium insufficiency is mitigated by increased calcium release from bones.

### 5.1. Patient Proficiency

Sufficient calcium intake is important for bone health, dental health, muscle function, and more [114,115,116,117]. Sufficient calcium intake is especially necessary in children and adolescents for the attainment of optimal peak bone mass. Sufficient calcium intake is also especially important in pregnancy, and to optimize both maternal and infant health [118].

Non-dairy sources of calcium include almonds, beans, blackstrap molasses, broccoli, dark leafy greens, bok choy, kale, collard, mustard or turnip greens, dried figs, okra, tahini, tofu, and tempeh. Fortified packaged foods, including plant milks, often contain added calcium. Absorption rates of plant calcium sources are affected by fiber and other compounds that diminish its total availability [119].

### 5.2. Practitioner Proficiency

The parathyroid gland, via the secretion of parathyroid hormone (PTH), tightly controls blood and interstitial calcium concentrations to ensure normal muscle contraction, nerve impulse transmission, coagulation function, and hormone secretion [120]. Insufficient dietary calcium intake results in decreased blood calcium concentration, which in turn results in increased parathyroid hormone secretion with two important effects. First, vitamin D is hydroxylated into its active form 1,25-dihydroxyvitamin D (calcitriol) [120]. This results in decreased urinary excretion and increased intestinal absorption of calcium. Second, PTH secretion results in the release of calcium and phosphate from bone. Because insufficient dietary calcium intake is mitigated by release of calcium from bones, chronically insufficient calcium intake is one modifiable factor in excessive bone loss [119,121,122,123].

Vitamin D deficiency, hypochlorhydria of any cause, including the use of H2 blockers or proton-pump inhibitors, or the intake of foods rich in sugars, fiber, phytate, and oxalate may reduce the bioavailability of calcium [11,25,124,125]. Oxalic acid is found in chard, collard greens, rhubarb, and spinach. Phytic acid is found in grains, nuts, seeds, and vegetables. Sprouting, soaking, or germination of grains and seeds reduces phytate binding; and cooking and fermentation helps break down fiber, and supports bioavailability [126]. Probiotics with phytase can block calcium binding to phytic acid.

Diets low in calcium or persons with poor calcium absorption may result not only in decreased bone mineral density, but also muscle dysfunction including cramps and spasms. Insufficient calcium intake may result in brittle nails, coarse hair, confusion, depression, memory loss, hallucinations and delirium, neurotransmitter dysfunction, irregular heart rhythms, cardiovascular disease, increased cancer risk, dental disease, and paresthesias [127].

Controversially, vegans and vegetarians may have lower bone mineral density and vegans may have increased fracture risk than the general public [127,128,129]. However, persons on a plant-based diet who ensure a sufficient intake of calcium and vitamin D may not have a higher risk of total or site-specific fractures, bone loss, and diminished bone height and bone weight [129,130]. This means that plant-based diets, like all other diets, must be mindfully planned to ensure an adequate amount, variety, and bioavailability of calcium-rich foods, including the use of supplemental calcium and Vitamin D, if needed [131,132].

Additional testing options include an assessment of the zinc protoporphyrin/heme ratio or reticulocyte hemoglobin content.

## 6. Question Five: What Is Your Preferred Source of Zinc?

Zinc is the second most abundant trace element, yet nearly two billion individuals worldwide lack adequate zinc intake. Although overt deficiency is not generally seen, zinc intakes may be below optimal levels in many persons on a plant-based diet because its bioavailability from plant foods is lower than that from animal products [133,134].

### 6.1. Patient Proficiency

Zinc status is closely linked to immune status, digestive efficacy, bone health, mood regulation, and cognitive functions [133,134,135]. Grain, legumes, soybean, wheat germ, seeds, nuts, nutritional yeast, leafy and root vegetables contain small amounts of zinc [136,137].

### 6.2. Practitioner Proficiency

Zinc status is important for over 300 reactions, including stomach acid and insulin production [138], protein metabolism, osteoblast mineralization [139], heme synthesis [140], and cytosolic antioxidant activity. Zinc plays multiple important roles in the central nervous system as it impacts glutamatergic neurons and circuitry throughout the cortex, amygdala, and hippocampus, regulates NMDA receptors and neuronal metabolism, and supports BDNF production and neuroplasticity [141,142,143]. Zinc impacts thyroid hormone activation [144], insulin action [138], leptin management [145], melatonin synthesis, gastrointestinal function and repair [146,147], and immune function [137,148,149,150].

Zinc levels influence vitamin A absorption, transport, and utilization [151]. Zinc deficiency may mimic iron deficiency symptoms [152]. Deficiencies can be seen in impaired health of skin, tongue, hair, nails, and eyes. Deficiency can be experienced as the loss of sense of taste or smell, delayed wound healing, impaired immunity, appetite loss, and growth retardation [133,134,135]. Zinc deficiency may not be demonstrable in blood tests [153].

Zinc deficiency concerns men and women in family planning stages due to higher risks of infertility and pre-eclampsia. Fetal complications can include congenital malformations, low birth weight, and growth retardation. Infant complications follow from the increased risk of premature birth including with retinopathy, necrotizing enterocolitis, and chronic lung disease [145,154]. Zinc is critical for male health in all ages for testosterone, prostate, sexual health, and reproduction [133,134,135].

Plant-based diets may increase the amount of zinc required in the diet due to the reduced bioavailability of zinc and is related to the high phytate content of many vegetable products. Well-planned vegetarian diets must be implemented to avoid dietary zinc deficiency and compensate for phytates and fiber absorption inhibitors [2,103,151,155,156,157,158].

Zinc bioavailability is increased with soaking, sprouting, and fermenting. Consumption of garlic and onions may help improve the absorption of dietary zinc [159]. Zinc absorption is moderately inhibited by the dairy protein casein. Non-dietary risk factors can increase zinc requirements. These factors include severe stress, alcoholism, diabetes, malabsorption, bariatric surgery, heavy perspiration, high-dose folic acid supplementation, and cadmium, copper or iron excess [135,155,157,158]. Appropriate assessment is needed to ensure adequacy in a plant-based diet.

## 7. Question Six: What Is Your Preferred Source of Vitamin D?

Vitamin D deficiency is a major global public health problem even in sunny equatorial countries. Musculoskeletal concerns with vitamin D deficiency include rickets, osteomalacia, osteoporosis, chronic musculoskeletal pain, muscular weakness, non-traumatic fractures, as well as falls [160]. Vitamin D deficiency has also been strongly linked to the incidence and severity of numerous extra-skeletal concerns, including immune, auto-immune, and infectious diseases, as well as numerous pregnancy concerns including gestational diabetes and pre-eclampsia [161,162].

### 7.1. Patient Proficiency

Plant-based diets do not provide vitamin D. The one exception is that 100 g of sun-exposed mushrooms can provide the RDA of 400 international units (IUs) of ergocalciferol (vitamin D2) [163].

This means that many persons on plant-based diets must rely on adequate sun exposure or supplementation. However, limited seasonal sun exposure in northern latitudes, indoor work and lifestyles, and the use of sunscreen, mean that many are at risk for inadequate vitamin D [164]. Vitamin D cannot be made when one is behind glass [165]. Furthermore, the more melanin in one’s skin [166], the higher one’s body mass index (BMI) [167], or the older one is [168], the more sun exposure one needs to make vitamin D. Vitamin D requirements are higher during pregnancy and lactation [169,170].

### 7.2. Practitioner Proficiency

Vitamin D is actually not a vitamin, but a seco-steroid hormone derivative of cholesterol that binds to nuclear receptors found in nearly every tissue in the body, including skeletal muscle cells, pancreas beta-cells, immune cells, and the placenta. Vitamin D binding to the nuclear receptor results in the up- or down-regulation of numerous genes that goes beyond the regulation of calcium and phosphate metabolism. These include regulation of cellular proliferation, differentiation, and apoptosis, plus innate and adaptive immunity [161].

Numerous studies have linked low vitamin D states with all-cause mortality, cardiovascular disease, numerous cancers including colon and breast, inflammation, disordered glucose metabolism and elevated lipid status, infectious diseases, autoimmunity including thyroid disease, rheumatoid arthritis and multiple sclerosis, mood disorders, declining cognitive function, and impaired physical functioning including falls [163,171,172,173,174,175,176,177,178,179]. Vitamin D deficiency is a major global public health problem even in sunny equatorial countries. Musculoskeletal concerns with vitamin D deficiency include rickets, osteomalacia, osteoporosis, chronic musculoskeletal pain, muscular weakness, non-traumatic fractures, as well as falls [180]. Vitamin D deficiency has also been strongly linked to the incidence and severity of numerous extra-skeletal concerns, including cardiovascular and cerebrovascular disease [162], chronic pain [181], immune [182,183], auto-immune [184,185,186], and infectious diseases [187], as well as numerous pregnancy concerns including gestational diabetes and pre-eclampsia [188,189,190]. Once orally absorbed, vitamin D2 or D3 is converted in the liver to 25-hydroxyvitamin D [25(OH)D], which is the best form to measure for assessment of vitamin D status [191]. This form is then converted once more into the active form, 1,25-dihydroxyvitamin D (calcitriol) by the kidneys. Liver or kidney dysfunction may impair vitamin D status.

The most common form of vitamin D found in over-the-counter supplements is D3 (cholecalciferol). This may be labeled as either vitamin D or vitamin D3. Cholecalciferol is commercially made from sheep lanolin exposed to ultraviolet B (UVB). This is bio-identical to the vitamin D3 synthesized endogenously in human skin with sun exposure. Vitamin D2 (ergocalciferol) is synthesized from UVB-induced transformation of the ergosterol found in the cell walls of mushrooms [163]. The avoidance of animal-derived vitamin D may be important for some. Vitamin D3 has a significantly longer half-life than D2 and is preferable over vitamin D2 for raising serum 25(OH)D levels [172,179].

The Endocrine Society’s guidelines define Vitamin D deficiency, insufficiency, and sufficiency as serum concentrations of 25(OH)D of <20 ng/mL, 21–29 ng/mL, and 30–100 ng/mL, respectively [177]. However, vitamin D function also depends upon magnesium status as a cofactor for vitamin D biosynthesis, transport, and activation [191,192,193].

### 7.3. Clinical Testing Notes

The one test for assessment of vitamin D status is 25-hydroxyvitamin D [25(OH)D] [191].

## 8. Question Seven: What Are Your Preferred Sources of Complete Proteins?

Patients who adopt a plant-based diet and health professionals who guide them must ensure the adequate intake of total protein in addition to the sufficient intake of the nine essential amino acids.

### 8.1. Patient Proficiency

All protein in the diet and all proteins in muscle, bone and brain are made from amino acids. Twenty amino acids exist and nine of these amino acids are termed essential. This means that they must be eaten as they cannot be made by the body. All 20 amino acids can be found in a plant-based diet. However, plants provide a less optimal balance and amino acid distribution compared to animal foods [194,195]. Persons consuming a plant-based diet need to ensure adequate total intake of protein as well as the complete range of essential amino acids [196,197].

Plant-based foods that contain all nine of the essential amino acids in themselves are infrequent. These include buckwheat, chia seeds, original Ezekiel bread, nutritional yeast, and quinoa. Daily meals should include mixed combinations of proteins to ensure the completeness of amino acid intakes. Common examples of complementary proteins include beans and brown rice, roasted vegetables and lentils, or different colored vegetables in a soup/stew with miso/lentils/beans and a whole grain. Protein digestibility of cereals, legumes, nuts, and seeds may be improved by soaking, sprouting, cooking, and, especially, pressure cooking [196,198]. Current data suggests that vegetarians consume adequate protein, but in a small number of vegans, there may be a modest risk of insufficient intake [199].

### 8.2. Practitioner Proficiency

Individuals on a plant-based diet are able to consume adequate protein. However, even persons on a relatively high-protein plant-based diet can be at risk for low intake of three essential amino acids, i.e., lysine, methionine and tryptophan [194,199]. Historically, an extreme example of an adverse consequence is kwashiorkor, the severe protein malnourishment disease that can occur despite a high-protein diet. This now rare condition was due to low lysine and tryptophan intake associated with a primarily maize-based diet [200].

Many plant-based foods can be great protein sources, but they vary considerably in their protein content as well as their amino acid content. Red flags for symptomatic patients are plant-based diets that are low in total protein; or that consist of a low intake of legumes, grains, seeds, or nuts. For example, one cup of quinoa contains 8 g of protein, whereas one cup of cooked lentils contains 18 g. However, unlike the complete amino acid profile for quinoa, lentils have very little of the essential amino acids’ methionine and tryptophan. They are also low in the amino acid cysteine. Thus, a diet containing predominantly lentils may be high protein and still be incomplete. Similarly, diets with high intakes of powdered plant-proteins may be incomplete. For example, commercially-available powdered pea and soy proteins have very low methionine content [201,202,203].

#### 8.2.1. Lysine

Diets with too few legumes, such as low lectin diets, may mean an insufficient intake of lysine. Significant lysine deficiency can exist despite a diet rich in fruits and vegetables, nuts and seeds, as well as corn, wheat, and brown rice [204]. Lysine insufficiency is a causal factor for delayed growth, insufficient collagen production, osteoporosis (due to urinary calcium loss), as well as recurrent herpes simplex outbreaks. Plant-based foods with higher amounts of lysine include avocados, wheat germ, and legumes (soybeans, beans, peas and lentils) [205].

#### 8.2.2. Methionine

Likewise, plant-based diets low in grains, such as gluten-free diets, but still rich in legumes, may be low in methionine. Commonly eaten foods low in methionine are asparagus, beets, broccoli, cabbage, carrots, cauliflower, celery, kale, pea, soy, spinach, squash, sweet potatoes, seaweed, turnips, and zucchini. Plant-based diets are best balanced with the inclusion of grains rich in methionine: wheat, wheat germ, millet, barley, rice including brown rice, corn, kamut, oats, rye, sorghum, teff, triticale, and quinoa. Of these, only millet, rice, corn, sorghum, teff, and quinoa are gluten-free. Oats, depending upon processing, may be gluten-free.

Methionine sufficiency is essential for growth, healthy hair, skin, and nails. Methionine sufficiency is required for selenium and zinc absorption, as well as T-cell proliferation and differentiation. Additionally, methionine is essential for methylation, an important factor in the prevention of both neural tube defects and osteoporosis [206,207]. Of note, patients may intentionally restrict methionine intake for health reasons, including life extension, fat loss, and cancer-cell-growth inhibition [208,209]. 

#### 8.2.3. Tryptophan

Tryptophan deficiency can occur in diets low in total protein or with low intake of seeds, (pumpkin, chia, sesame, sunflower, and squash), nuts, legumes, and grains. Concentration of this essential amino acid in proteins is most often significantly lower than for other amino acids. For example, plant-based diets rich in foods, such as lentils, avocados, broccoli, eggplant, spinach, and tomatoes, may be low in tryptophan [203,210].

Tryptophan is the essential amino-acid necessary for growth, as well as the production of serotonin, melatonin, and vitamin B3 (niacin, nicotinamide). Tryptophan deficiencies can present as mood disorders, fatigue, insomnia, or disordered eating. Vitamin B3 deficiencies can present with headaches and dizziness, as well as changes in skin, mood, energy, cognition, and digestive system function [211]. (Of note, symptoms of tryptophan deficiency may occur at intakes as little as 25% below the required daily intake [211].)

#### 8.2.4. Conditionally Essential Nutrients

Persons on plant-based diets are at risk for insufficient levels of three conditionally essential molecules that are readily found in omnivore diets: 1) creatine (from methionine, glycine, arginine), 2) carnitine (from methionine, lysine), and 3) taurine (from methionine, cysteine). These conditionally essential nutrients, under normal conditions, can be produced by the body in amounts sufficient to meet physiological requirements. However, pathophysiological stress and/or restricted dietary intake of key amino acids can impair their production and increase the risk of multiple medical concerns.

#### 8.2.5. Creatine

Creatine supplies energy to both the muscle and brain to meet suddenly increased energy demands. Plant-based diets are inherently low in creatine, a principal component of both muscle and brain. Supplementation may be important. For persons with a genetic impairment of creatine production, creatine supplementation reverses cognitive and neurodevelopmental defects [212]. Similarly, creatine supplementation has resulted in improved memory in young female vegetarians, and short-term memory and intelligence/reasoning in other normal populations [213,214]. Creatine supplementation in vegetarian athletes has resulted in significant improvements in both muscular strength and endurance in addition to memory [215].

#### 8.2.6. Carnitine

Carnitine status is clinically relevant as it is crucial for mitochondrial function and cellular energy production. Persons consuming a plant-based diet under increased physical stress, including pregnancy or dialysis, are at increased risk of hypocarnitinemia [212,216]. Carnitine shuttles long-chain fatty acids across the mitochondrial membrane from the cytosol into the mitochondrial matrix for β-oxidation. Carnitine production is especially important for cardiac and skeletal muscle function. Moreover, carnitine has anti-inflammatory and antioxidant properties and plays a role in insulin sensitivity [217].

Defective fatty acid oxidation can present as fatigue, non-alcoholic fatty liver disease, fat accumulation, or hypoglycemia [218,219,220]. Fatty acid oxidation is also important for oocyte developmental competence [128]. Patients with chronic diarrhea or taking valproic acid, omeprazole, or zwitterionic drugs such as levofloxacin are at risk for secondary carnitine deficiency [221].

#### 8.2.7. Taurine

Taurine is one of the most important amino acids in high energy tissues, including the brain, retina, and muscles. Plant-based diets do not contain taurine [222]. Analysis of taurine levels in vegans have been slightly lower to half that of omnivores [223].

Taurine is critical for neurological development and neurodegenerative protection. In the eye, taurine provides critical antioxidant protection for the retina, lens, vitreous, cornea, iris, and ciliary body. This includes critical photoreceptor and retinal pigment epithelium antioxidant protection [224,225].

Taurine supports bile flow and cholesterol conjugation. It affects blood pressure regulation, mitochondrial function, and electron transport chain activity [224,225]. Taurine provides valuable xenobiotic detoxification and protects against degenerative endocrine hormone disruption [226].

Consequences of inadequate taurine intake or production include magnesium wasting, cardiomyopathy and arrhythmias, renal dysfunction, pancreatic beta cell dysfunction, plus numerous neurological and vision disorders including loss of retinal photoreceptors. Additional concerns with insufficiency include infertility, still births, and neonatal developmental problems [224,225].

#### 8.2.8. Protein and Energy Intake during Caloric Restriction

The RDA for dietary protein intake is set at 0.8 g/kg of body weight [227]. Free-living individuals consuming meat report consumption of 17% energy as protein, while those on a vegan diet report ~13% [21]. At an energy intake appropriate for weight maintenance, this level of protein is adequate. However, when individuals consume a low-calorie diet, whether on purpose or due to medical or psychological factors, special attention must be paid to maintain protein intake based on body weight. Reducing energy intake without attention to total protein intake on a total plant-based diet may result in insufficient intake of protein and amino acids.

The value of dietary protein depends not only on amino acid composition, but factors such as the protein digestibility corrected amino acid score (PDCAAS) [199,228]. PDCAAS is a complex indicator of protein quality used to assess the capacity of dietary protein to meet amino acid requirements in the diet [229,230]. For example, one gram of protein with a low PDCAAS does not provide the same amino acid protein bioavailability and content as one gram from a high PDCAAS food. Beans and legumes have a PDCAAS score of 45–70 percent and rice provides a PDCAAS score of 56–62 percent. In contrast, processed soy as soy protein isolate provides 100% of the PDCAAS. Lower PDCAAS scores are one factor dieticians consider for the maintenance of muscle mass in obese-sarcopenic, elderly-sarcopenic, or athletic patients [196,197,201,231,232].

Additionally, protein digestion and absorption are adversely affected by the numerous causes of hypochlorhydria, including aging, medications, and gastric bypass/banding. Furthermore, the phytic acid and trypsin inhibitors found in many plants may impact protein digestibility. Humans may lack adequate phytate-degrading enzymes in the digestive tract and the body may require increased dietary protein in order to compensate. Phytic acid is found in cereals, legumes, seeds, and nuts. Trypsin inhibitors are found in common beans, chickpeas, lentils, peas, broad beans, peanuts, and soybeans. Phytic acid and trypsin inhibitors may be reduced by soaking and cooking these foods [135,233]. The inclusion of probiotic supplementation may enhance blood levels of amino acid levels from incomplete plant proteins [234].

### 8.3. Clinical Testing Notes

Assessment of specific amino acid distributions in the diet and of total protein intake is primarily based upon patient self-report. In children, height and weight should be measured at each clinical visit. Symptomatic patients for any reason will likely benefit from the screening for blood urea nitrogen, creatinine, total protein, and albumin found in commonly ordered metabolic profiles.

## 9. Conclusions

Intentional plant-based diets provide numerous health benefits. Yet, if poorly informed, combined with restrictive diets, or combined with certain prescription medications, they can drive or worsen physical symptoms. Furthermore, well-informed practitioner attention is required with the special situations of pregnancy, breastfeeding, childhood/adolescence, intense physical work, athletic training, and concomitant medical conditions. As with all medical interventions, physicians must be aware of the benefits, risks, and potential concerns following from prescribed plant-based diets. In addition, with more patients self-initiating plant-based diets, all practitioners need the capacity to question, assess, test, counsel, and appropriately refer for nutritional concerns. The seven questions identified here provide a framework for the rational consideration of several nutritional factors that may underlie a symptomatic patient’s presentation. They can be incorporated into pre-visit questionnaires or into visits.

One limitation in this review is the exclusion of questions regarding dietary sources of riboflavin (vitamin B2), choline, iodine, and selenium or other nutritional deficiencies. These remain important medical concerns, but represent emerging areas of clinical interest with limited research data for persons on plant-based diets. Likewise, further scientific study is required to understand the value of plant ferritin as a source of iron and endogenous cobalamin synthesis as a source of vitamin B12. Moreover, not addressed above is the potential for iatrogenic vitamin K deficiency for persons on a plant-based diet prescribed vitamin K antagonists (i.e., Coumadin) for anti-coagulation with venous thromboembolism, atrial fibrillation, and prosthetic heart valves. In the past, physicians have ordered patients to not consume vitamin-K-rich foods, such as green leafy vegetables [235,236]. However, the evidence for the avoidance of vitamin-K-rich foods is weak and persons on a plant-based diet, with appropriate INR monitoring, do not need to avoid a plant-based diet [237].

Health professional competence increasingly requires a working knowledge of clinical nutrition. This review, and the seven questions identified here, support clinician capacity to include nutritional concerns into the differential diagnosis for any given presenting patient concern. The underlying hypothesis is that clinician consideration of potential nutritional insufficiencies will guide both appropriate diagnoses and cost-effective treatments. For further guidance, laboratory tests are found in Table 1, and several helpful plant-based references for clinicians and patients may be found in Table 2.

## Figures and Tables

**Table 1:** Helpful resources for a healthy plant-based diet (sites accessed 9 April 2020).

Kaiser Permanente. The Plant-Based Diet: A Healthier Way to Eat https://thrive.kaiserpermanente.org/care-near-you/southern-california/center-for-healthy-living/wp-content/uploads/sites/30/2017/12/Plant-Based-Diet-Eng.pdf
Kaiser Permanente. Plant-Based Eating. Eat Healthy, Live Better https://1w7lsd145y0×22fgi71wekk2-wpengine.netdna-ssl.com/wp-content/uploads/15116-plant-based-eating.pdf
Physicians Committee for Responsible Medicine. Good Nutrition. https://www.pcrm.org/good-nutrition
Physicians Committee for Responsible Medicine. Nutrition Guide for Clinicians. https://nutritionguide.pcrm.org/nutritionguide/view/Nutrition_Guide_for_Clinicians/1342057/all/Micronutrients_in_Health_and_Disease#2
USDA National Agricultural Library. Food and Nutrition Information Center. Information for Health Professionals. https://www.nal.usda.gov/fnic/information-health-professionals
USDA National Agricultural Library. Food and Nutrition Information Center. Dietary Guidance. https://www.nal.usda.gov/fnic/dietary-guidance-0
USDA Nutrition Evidence Systematic Review. https://nesr.usda.gov/
USDA Choose My Plate. Tips for Vegetarians. https://www.choosemyplate.gov/node/5635
US National Library of Medicine. MedlinePlus. Vegetarian Diet. https://medlineplus.gov/vegetariandiet.html
Forks over Knives Tools. https://www.forksoverknives.com/tools/#gs.2v5u3g

**Table 2:** Clinical Tests Relevant to Nutritional Assessment.

Clinical Tests	
**Protein**	Total serum protein Serum albumin Prealbumin Globulin Retinol-binding protein Creatinine BUN Additional specialized tests may be warranted [238,239].
**Vitamin B12**	Fasting serum B12 (12 h) Holotranscobalamin or vitamin-B12-binding capacity, unsaturated Total plasma homocysteine Methylmalonic acid Mean corpuscular red cell volume Parietal cell antibodies Intrinsic factor antibodies
**Iron**	Serum ferritin Total iron, iron-binding capacity, % saturation (calculated)
**Fatty Acids**	Omega-3 (EPA + DHA) index Omega-6/omega-3 ratio EPA/Arachidonic acid ratio Arachidonic acid EPA DHA
**Calcium**	Serum calcium RBC Calcium Parathyroid hormone Serum 25-hydroxyvitamin D C-terminal telopeptide of type-I collagen (CTX-I)
**Zinc**	Serum or RBC zinc Serum copper Serum ferritin
**Vitamin D**	25-hydroxyvitamin D, immunoassay 25-hydroxyvitamin D (D2, D3), LC/MS/MS

## Data Availability

Not applicable.
